# Work station learning activities: a flexible and scalable instrument for integrating across basic subjects in biomedical education

**DOI:** 10.1186/s12909-017-1084-z

**Published:** 2017-11-29

**Authors:** Rocío González-Soltero, Ana Isabel R. Learte, Ana Mª. Sánchez, Beatriz Gal

**Affiliations:** Departamento de Ciencias Biomédicas Básicas, Facultad de Ciencias Básicas y de la Salud. Universidad Europea de Madrid, Calle Tajo s/n, 28670 Madrid, Villaviciosa de Odón Spain

**Keywords:** TBL, WSLA, Integrated curriculum, Biomedical schools

## Abstract

**Background:**

Establishing innovative teaching programs in biomedical education involves dealing with several national and supra-national (i.e. European) regulations as well as with new pedagogical and demographic demands. We aimed to develop and validate a suitable instrument to integrate activities across preclinical years in all Health Science Degrees while meeting requirements of national quality agencies.

**Methods:**

The new approach was conceived at two different levels: first, we identified potentially integrative units from different fields according to national learning goals established for each preclinical year (national quality agency regulations). Secondly, we implemented a new instrument that combines active methodologies in Work Station Learning Activities (WSLA), using clinical scenarios as a guiding common thread to instruct students from an integrated perspective. We evaluated students’ perception through a Likert-type survey of a total of 118 students enrolled in the first year of the Bachelor’s Degree in Medicine.

**Results:**

Our model of integrated activities through WSLA is feasible, scalable and manageable with large groups of students and a minimum number of instructors, two major limitations in many medical schools. Students’ perception of WSLA was positive in overall terms. Seventy nine percent of participants stated that WSLA sessions were more useful than non-integrated activities. Eighty three percent confirmed that the WSLA methodology was effective at integrating concepts covered by different subjects.

**Conclusions:**

The WSLA approach is a flexible and scalable instrument for moving towards integrated curricula, and it can be successfully adapted to teach basic subjects in preclinical years of Health Science degrees. WSLA can be applied to large groups of students in a variety of contexts or environments using clinical cases as connecting threads.

## Background

Spain is one of 29 European countries that signed the Bologna Declaration in 1999, which laid the foundation of the European Higher Education Area (EHEA). Its aim was to make higher education more relevant to the individual, allowing students to easily move between degrees and countries, and from academia to job market [1]. The Bologna Process has driven strong educational reforms at the national level. To articulate this change in Spain, new laws were published in 2007 and the national quality assessment agency (ANECA) was created. ANECA follows EHEA instructions to refocus curricula on acquiring skills and learning outcomes. Under ANECA’s auspices, an integrated curriculum was recommended as the best way to achieve the acquisition of competences, thus prompting Spanish universities to adopt the required changes in teaching methodologies with an increase in active learning activities at the expense of teacher-centered lectures. This point was specially highlighted for biomedical education by the signatories of the Granada Declaration back in 2001, where achieving horizontal and vertical integration of subjects was a matter of concern for policymakers. However, despite these national recommendations, and that medical education reformers advocated combining disciplines and organizing integrated learning experiences for students, the influence of a Flexner vision of biomedical education [2, 3] has been such that most curricula still retain a basic non-integrated teacher-centered model with few active-learning sessions [4]. Under a Flexnerian vision, disciplines were taught separately with an emphasis on basic sciences in the early years and clinical experiences in the later years. Since the time of Flexner [3], the medical school basic science curriculum has largely consisted of discrete courses controlled by individual departments.

With this scenario, curricular integration has become a difficult and complex task. In some leading institutions, however, integration approaches follow Harden’s model [5]. According to this model, integration is achieved by interrelating and unifying subjects that are frequently taught across separate courses and/or departments. Harden illustrates the integration process as a ladder that represents the different levels of curriculum integration, with up to 11 steps. At the highest level, the curriculum is totally integrated and teaching is active and student-centered. The objective is that students gain competences as they learn. Thus, they are provided not with a theme or topic, but with a scenario reflecting the field of knowledge closest to their future professional life [6]. Basic science content, for example, is considered in the context of clinical medicine. This solves a longstanding major problem of traditional curricula: that of students failing to see the relevance of what is taught to their future career as doctors [7].

Using Harden’s approach also implies adopting small-group teaching so that active participation and teamwork skills can be developed while learning by doing [8]. Here, instructors become more aware of students’ knowledge and skills so that they can facilitate understanding of complex information typical of basic sciences with an integrated view [9]. Small-group teaching methodologies include Problem Based Learning (PBL), Case Based Learning (CBL) and Team Based Learning (TBL). One of these approaches, TBL, has become a major instrument in many Health Science schools. TBL merges individual and group learning approaches, with the instructor playing a supervisory role through comments and question rounds. It fits excellently with the current paradigm; it prepares students to manage in a realistic step-by-step scenario while working in a collaborative way and learning how to do it by themselves [9, 10]. Vasan wrote in 2009 [11] “as medical schools are creating integrated and interdisciplinary courses during the preclinical years, team-based learning is particularly useful because of its emphasis on teamwork, mastery of content, and problem solving for clinical application”. Here, specific pedagogical factors, namely learning objectives and methodological aspects, are critical variables for successful integration with TBL [12]. Evidently, adopting such approaches requires a larger faculty at a time when universities face new demographic pressures.

To achieve these pedagogical goals, while also complying with national and European regulations, the Department of Basic Biomedical Sciences at Universidad Europea de Madrid (UEM) has embarked on a curricular review that redefines teaching to horizontally integrate basic subjects within the preclinical years of our health sciences degrees (Medicine, Dentistry, Pharmacy, Biotechnology, and Nursing). Under these conditions, we have implemented a new instrument based on active-learning approaches to delineate scalable modules of Work Station Learning Activities (WSLA), Here, we describe our approach and test preliminary results of our pilot experience to lay the foundations for new curricular actions aimed at achieving horizontal and vertical integration of basic and clinical sciences within Health Science undergraduate degree programs.

## Methods

### Designing integration modules: The role of the integration subcommittee

Table 1 synthesizes some facts and steps on the evolution towards the integrated curriculum of Medicine degree at UEM.

**Table 1:** Facts and steps on the evolution towards an integrated curriculum of Medicine degree at UEM

Bachelor’s Degree in Medicine at UEM	
• Six-year program with two preclinical years.	
• Preclinical courses are covered by the Department of Basic Biomedical Sciences.	
• First preclinical year includes our courses of Anatomy, Physiology, Cell Biology, Genetics, Biochemistry and Histology.	
• Second preclinical year includes our course on Structure and Function of Organs and Systems.	
• Subject organization is based on 70% lectures, 10–15% practical sessions and 10–15% integrated cases activities (within the integrated WSLA module).	
Evolution of the Integration Course Module	
• 2005 The Structure and Function Project kicks off.	
• 2009 Establishment of the Structure and Function module in our Bachelor’s Degree in Dentistry.	
• 2010 Establishment of Structure and Function of Organs and Systems in our Bachelor’s Degree in Medicine	
• 2015 First series of Structure and Function Integration Workshop.	
• 2016 Implementation of the Integrated WSLA Module in the preclinical courses- Bachelor’s Degree in Medicine.	

The present study describes a pilot program that implements our new instrument, namely the Work Station Learning Activities (WSLA), for teaching basic sciences with a horizontally integrated scheme. With the purpose of designing our flexible approach, we first established the Integration Subcommittee to review, track and improve curricular integration, similar to other universities [13]. The Integration Subcommittee was formed by eight faculty members from different fields of knowledge (i.e., biology, physiology, genetics, histology, and biochemistry). Two additional specialists on pedagogy joined the subcommittee with the mission of guiding the process of curricular shifting from a pedagogical point of view. They all had autonomy and power to lead the curriculum reform beyond specific faculty interests. The Integration Subcommittee was responsible for identifying gaps and synergies between separate subjects and for selecting a set of learning objectives to be integrated. The Integration Subcommittee reported to our Dean and Academic Director, both of whom had been personally involved throughout the process.

Based on these top-down directives, our Faculty members then worked together to propose a different set of WSLA modules, as fully described in the Results section. Each WSLA module was conceived in accordance with a horizontal integration scheme to cover learning goals of basic sciences as identified by the subcommittee within the context of the Spanish national framework ANECA. Following a Harden’s ladder integration scheme, we pursued the sharing step at which several disciplines merge in a scalable teaching program of common individual learning objectives. We actively looked for integrated sessions to be considered important as independent subjects themselves, in terms of time, resources and assessment [5, 14]. As described later in Results, these definitions were critical in preparing each WSLA module. The direction, support and supervision of the Integration Subcommittee continued throughout this bottom-up process by meeting once a week with the faculty members involved in designing and implementing each WSLA module.

### Sample and survey description

We applied our WSLA instrument in early preclinical courses of the Bachelor’s Degree in Medicine during the academic year 2015–2016. To evaluate students’ perception of WSLA, a total of 118 students enrolled in the first year of the Medicine degree were selected for a survey study. The survey, consisting of four questions (seven items) split into two blocks, was supervised by an independent group of experts including psychologists and specialists in medical pedagogy. The first block of questions aimed to evaluate statements regarding a particular WSLA module. The second block was used to evaluate different environments in which WSLA was implemented: laboratory practices, lectures, and gamification sessions. A similar survey was previously published as part of an ongoing longitudinal project [15], and was based on previous studies [16]. Questions had to be answered following a five-level Likert scale (ranging from strongly disagree, 1, to strongly agree, 5). A cumulative proportion of the students who were in agreement (4–5) or disagreement (1–2) was determined. The survey was approved by the Integration Subcommittee and by the Ethics Committee of our university (CIPI/071/17).

### Statistical analysis

Survey data were analyzed using SPSS Software (SPSS for Windows, Version 17.0, SPSS Inc., Chicago, IL, USA). Microsoft Excel (Excel, Microsoft Corporation) was used for data presentation. The mean value and standard deviation were determined for each parameter. Student’s t-Test for independent variables was used to investigate statistically significant differences between the means of various datasets. An ANOVA was used to investigate differences regarding the students’ support for specific teaching methods.

## Results

### Development of WSLA integrated modules

**Fig. 1:**
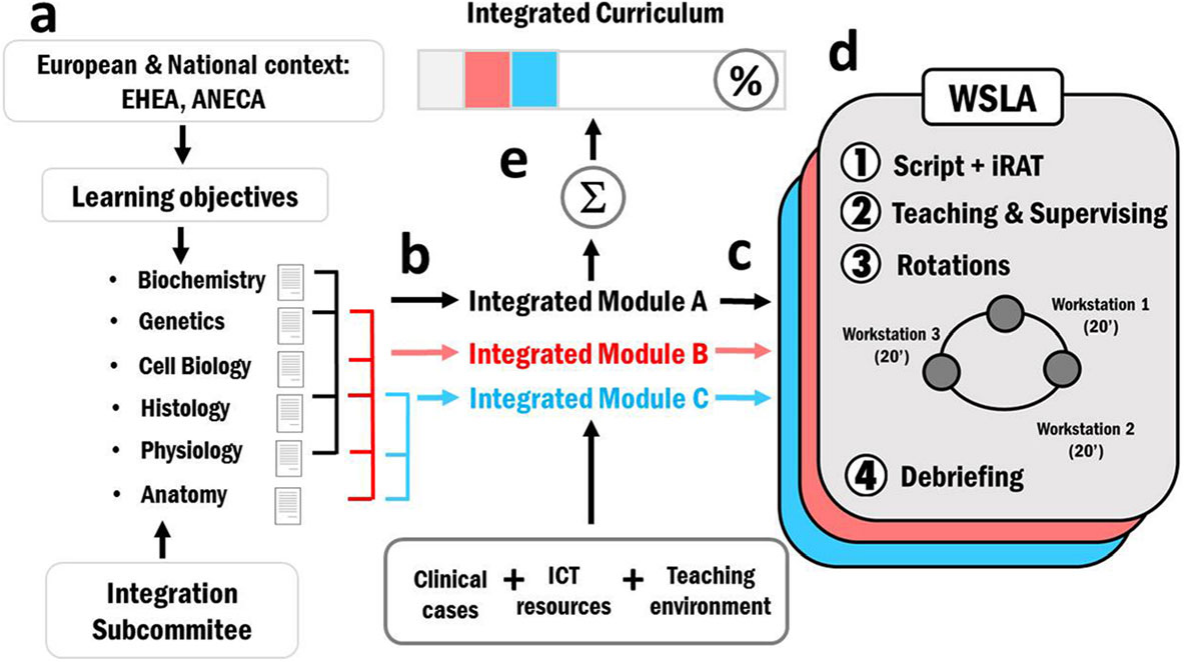
Designing WSLA modules. Schematic representation of the different phases leading to the implementation of WSLA modules. The European Higher Education Area (EHEA) and national agencie (ANECA) determine the regulatory context as well as skills and learning objectives for Medical Education. First (**a**), an Integration Subcommittee identifies a set of learning objectives that could be potentially integrated across subjects. Next, faculty members describe the learning objectives suitable for a particular integrated module, like for example pH control which can integrate objectives from Biochemistry and Physiology. Each integrated module can target different subset of objectives (i.e. A, B and C identified with different colors in the scheme) (**b**). Then, each module is conceptualized in the best teaching environment (i.e. laboratory, simulated hospital, etc.…) by identifying the best clinical case, as well as the more suitable information and communication technologies (ICT) plus other resources to be used (**c**). This yields to the final configuration of WSLA for each particular module (**d**). Each WSLA involve a detailed script and individual Readiness Assurance Tests (iRAT). Rotations along different workstations proceed under the supervision of teachers. Each WSLA terminates with a debriefing session. Our instrument is flexible and scalable; several WSLA modules could be combined (color coded WSLAs) to contribute the curricula integration achievement. The more WSLA modules are implemented the larger the integration level of the curriculum (**e**)

In designing one WSLA module, a subset of learning objectives suitable for being integrated among the different subjects was first identified by faculty members of a particular discipline from those provided by the Integration Subcommittee (Fig. 1a). Each of these learning objectives is described independently in the syllabus of the different subjects covering that unit. Each WSLA module will seek to reinforce integration of those objectives, so that students can understand them together. For instance, learning objectives for pH control, which is traditionally taught separately in physiology and biochemistry, read as follows:

1. To understand the concepts of pH, pKa and chemical buffer and their application in Medicine.
2. To define the normal range of pH in body fluids and the concepts of acidosis and alkalosis.
3. To integrate the concept of pH in the context of cell physiology, understanding how different carriers (for example, the Na^+^/H^+^ exchanger, the Cl^−^/HCO3^−^ exchanger, the Na^+^/HCO^3−^ cotransporter) contribute to the control of pH.
4. To identify the mechanisms of respiratory and renal compensation caused by increase or decrease of pH in body fluids.

In this example, all these learning objectives will be integrated tightly together in a dedicated WSLA pH module (Integrated module A in Fig.1b). Next, we looked for an appropriate combination of active educational resources and the most suitable teaching environment to fit a clinical scenario in workstations, using one clinical case as a common thread (Fig.1c). Here, a workstation is defined as a work area with access to some educational resources through which students could self-explore aspects of learning objectives in each module under teacher supervision. For instance, the use of medical imaging is paramount to radiology, cardiology and internal medicine, and medical imaging laboratories are supported by heterogeneous computer systems as well as anatomical models and other data sources [17]. A combination of these tools has been used before to self-direct learning in anatomy [18]. Thus, we built up each WSLA around the concept of workstations within a particular clinical case so that each integrated module meets learning objectives of intermixed basic subjects. Workstations are conceived so that each mimics a particular aspect of a real case scenario using gamification, dissecting rooms, the simulated hospital, virtual microscopy devices, radiology images and 3D anatomical atlases as resources across which students rotate (Fig.1d). For the WSLA pH module, for instance, the clinical case was aspirin intoxication [http://sciencecases.lib.buffalo.edu/cs/collection/detail.asp?case_id=498&id=498]. Workstations were situated in the practice laboratory as follows: workstation 1 with material to run an experiment related to the variation of pH; workstation 2 with computer tablets including the ADAM interactive Physiology Apps [http://www.adameducation.com/interactivephysiology]; workstation 3 with anatomical models and physiology books.

We followed a modified scheme based on a TBL strategy adapted to a 2-h session. Each module included a first step in which the student was provided with a script prior to each integrated case session and the Individual Readiness Assurance Test (iRAT) [10] (Fig.1d). The script depicts the clinical scenario, and covers general learning objectives, methodology, and the procedure and evaluation for each workstation. Besides this, there is specific material for each WSLA module that students have to prepare in advance so that they have all the material available in the virtual platform 1 week before the WSLA module takes place. In the WSLA pH module, for instance, the specific material consists of a PowerPoint presentation and a scientific bibliography on the concept of pH, to be read in advance. It also includes some open questions, schemes and pedagogical material to be used later for evaluation purposes once activities are completed. The iRAT is both a self-assessment and a knowledge test. It consists of a multiple choice test (typically 10–20 questions) that allows students to self-evaluate whether they have successfully integrated some previous knowledge required for running activities. On the activity day, the WSLA module starts by solving the iRAT in place to check whether students are ready to go through rotations (Fig.1d; step 1). In a second step, an instructor organizes the class in small groups (5–6 members in our case) and performs a clarification review of iRATs, presenting the case so that students can clarify doubts and clearly identify the concepts to be mastered at each workstation (Fig.1d; step 2).

During a third step, students will rotate within all workstations (20 min each) in each WSLA module, solving the main core problem represented in the clinical scenario presented (Fig.1d; step 3). Our WSLA instrument can be used with large groups of students and a minimum number of instructors. Usually we have one group of about 50 students running one WSLA module at a time (1 day), with about 5 groups in a week. With this design there are no more than about 20 students organized in small groups of 5–6 members at each workstation, so that we just need to scale resources accordingly. We usually have a ratio of one professor per 15 students, even though TBL can run with large classes (45–50 students) [19].

Despite being split into different workstations, understanding concepts at each of them requires students to envision the whole clinical scenario. This means that after completing all workstations, students review their integrative knowledge, interpretations and calculations. At each workstation, students have to complete an evaluation test to measure learning, which is then delivered to instructors. The material students work with at each workstation varies depending on the best resources to accomplish learning objectives for each integrated module, including open-short or multiple-choice questions, scientific calculations and plots, and competency-based assessment rubrics [https://www.ncbi.nlm.nih.gov/pmc/articles/PMC2931194/]. The last step of each WSLA module leads to a final simultaneous report on the clinical scenario wrapped up in a final debriefing (Fig.1d; step 4). The original script provided to students is then completed and, together with all aforementioned material, preserved to be used for individual study.

Our design is fully scalable by definition. In Fig. 1, different colors are used to identify individual integrated WSLA modules. The more WSLA modules, the more integrated the curricula (Fig.1e), thus allowing institutions to build up across Harden’s steps over time ([15, 20] http://www.gamification.co/2013/03/20/gamification-in-healthcare). Given its flexibility, we consider WSLA to be a teaching instrument that can easily be adapted to different educational contexts. Also, because of the bottom-up nature of WSLA design, it can be adapted to easily capitalize available institutional resources. Our new integrated teaching instrument currently represents about 10–15% of the teaching load in each preclinical year of the undergraduate program, similar to other Health Sciences degrees. We aim to target a fully integrated curriculum using this approach in forthcoming years.

### Evaluation of learning outcomes in WSLA modules

Evaluation of learning achievement is critical for any new teaching instrument and a method of evaluation should accompany our curricular innovation [14]. Each WSLA module is assessed by collecting questionnaires and material from different workstations, yielding a global score for each student within a team. In order to assess students’ success in achieving learning objectives during each WSLA, we considered the scores of workstation material, results from iRATs, and the overall assessment in a complete WSLA score for each student. In the pH WSLA for instance, students had to answer 15 multiple-choice and 12 reasoning questions followed by an open discussion session at the end to debrief pH learning objectives. Mean scores obtained from different WSLA for each student contribute to evaluate their practical performance and skills in the final score. To further reinforce evaluation of WSLA learning, questions regarding each WSLA are also included in the general examination that every student should undertake for each learning topic. For instance, in the final examination of the topic physiology and biochemistry we inserted five questions out of 40 regarding pH. Thus, results from each WSLA module contribute to both knowledge and skills evaluation. Overall the specific weight of integrated activities over the final student’s mark represents the percentage of the teaching load covered.

### Students’ perception of WSLA methodology in integrated learning

Data presented here refer to 118 first-year students enrolled on the Bachelor’s Degree in Medicine during academic year 2015–2016. They were asked whether they found WSLA more useful than simultaneous non-integrated activities they were participating in (question 1 in Table 2). The proportion of students that selected an answer in the agreement range (4–5) was 78.9% (cumulative agree). When asked whether they preferred traditional lectures to WSLA (question 2, Table 2), only 18.7% of students selected this option, whilst 24.6% exhibited no preference for a particular method of teaching. Participants were also asked to evaluate whether WSLA effectively integrated concepts across separate subjects: 82.9% of participants agreed in this case (cumulative agree) (question 3, Table 2).

**Table 2:** Survey Results (in %)

Survey questions regarding WSLA	Strongly agree (5)	Agree (4)	Indifferent (3)	Disagree (2)	Strongly disagree (1)	Mean value
1- I find WSLA activities more useful than simultaneous non-integrated activities	15.3	63.6	16.9	3.4	0.8	3.9
2- I would prefer traditional lectures rather than WSLA.	3.4	15.3	24.6	34.7	22.0	2.4
3- The WSLA activity has helped me to integrate several topics included in different subjects	41.9	41.0	12.8	1.7	2.6	4.2
4- Which of the following activities motivates you most in your learning experience?						
a) Traditional lectures	6.8	41.9	31.6	11.1	8.5	3,3
b) Gamification	20.5	27.4	29.9	12.0	10.3	3,4
c) Laboratory sessions	53.4	35.0	8.7	1.9	1.0	4,4

In our survey, students were also invited to identify their preferred teaching modality (question 4; Table 2). In the particular WSLA we were evaluating with the survey, gamification and laboratory sessions were exploited to define workstations [18]. Participants were asked to rate laboratory practices, lectures, and gamification sessions using a Likert scale from 1 to 5, 1 being the lowest score. Students gave laboratory practical sessions the highest rating (4.4 out of 5). Gamification and lectures were assessed similarly (3.4 and 3.3 out of 5, respectively). An ANOVA with repeated measures showed significant differences between the three teaching methods (*p* < 0.01), laboratory sessions being the preferred modality as tested with post-hoc tests (Table 2).

## Discussion

Here we have described a flexible and scalable teaching instrument, the WSLA, which is conceived as a horizontally integrated scheme for reaching Harden’s sharing and correlating integration levels across basic subjects in the early years of undergraduate degrees in the field of biomedical education. This new instrument offers several pedagogical and practical advantages for coping with national regulatory demands while advancing towards a fully integrated Harden’s model that has been recommended as an educational strategy [21]. Whereas integration was once regarded as a mark of innovation, it is now more widely accepted as a feature of all educational programs. Our WSLA approach has been designed to reach the sharing/correlation step of Harden’s ladder in a flexible and scalable way.

WSLA has many advantages as a teaching instrument. First, it allows for a progressive modification of the biomedical curriculum. As in many universities, our current Bachelor’s Degree in Medicine is divided into blocks representing the main basic subjects (anatomy, histology, physiology, biochemistry, cellular biology and genetics), which are taught according to a Flexnerian approach. Bearing in mind continual medical scientific advances and the recommendations made by national agencies, it is imperative to evolve towards more integrated competency-based curricula [14]. In Spain today, many of the new active learning methodologies are legally restricted and their application is especially hampered by the large number of students. Given its modularity, WSLA represents a flexible instrument that can be applied to large groups of students with a minimum number of instructors.

Secondly, because WSLA modules can be handled independently and coexist with classical lectures, the instrument is scalable to increasing levels of integration across years. This endows WSLA with a unique transformative capability. Our WSLA design has strong roots in TBL methodology. TBL allows for active learning in large groups with immediate feedback and a minimum allocation of instructors [10] [22]. Being student-centered, TBL also allows for a key leader role for instructors who define learning objectives [23]. By working in small groups at each workstation while interacting with instructors allows for a more personalized teaching. The latter was critical for our WSLA design, since large groups are less conducive to effective learning experiences. In WSLA, iRATs allow instructors to supervise knowledge, and results are included in the student’s advance assignment. By means of rotations, students are immersed in a Team Application Process (tAPP) from the outset, something close to a real clinical scenario where problems have to be solved fluidly in a team-based framework with no time for much individual thinking. Therefore, deductions are made by the group from the very beginning, and so completion of each workstation emulates a clinical environment. In this regard, WSLA helps students to develop critical thinking regarding multiple aspects of a unique clinical case, and to cope with mistakes [24]. In WSLA, clinical scenarios are reinforced by providing workstations with the best technological applications, always related to the case under study. Organizing such case threads in the format of workstations ensures that students assemble pieces of knowledge, relating concepts belonging to different knowledge fields to one particular problem to be solved in each session. This situation resembles more closely the one they will face once they embark on their professional career. It is worth mentioning that this takes place in a limited time frame, making its application feasible in different situations, and with a minimum investment in instructors and time.

Importantly, WSLA is flexible enough to incorporate and adapt other learning methodologies and curricular designs. For instance, WSLA can offer a useful approach to organize objectives into learning spirals [25]. In a spiral curriculum there is an iterative revisiting of topics throughout the course and years at different levels of difficulty, so that new learning is related to previous learning and the competence of students increases with each visit to a topic. Because each WSLA will pivot around clinical cases, they can be easily used as common threads to avoid redundancies and to promote integration at all desired levels.

Shifting curricula from fragmentation to integration is a major challenge that requires careful management by institutional administrators and faculty members. In our university, teachers have been receiving training since 2015, when the first Structure and Function Workshop took place (Table 1). The workshop consisted in selecting some demonstration units to be integrated, designing WSLA integrating modules and programming the new academic year. Since then, an annual workshop organized by the training department and the Integration Subcommittee is devoted to discussing the experience, updating material and approaches, and receiving timely training in close interaction with leading pedagogical experts in the fields [26]. Therefore, WSLA also have a strong impact on departmental dynamics by promoting training, interaction and cohesion between faculty members.

Regarding students’ perception of WSLA as a useful integration tool for learning, 79 % of participants stated that WSLA modules were more useful than traditional master classes, with a majority acknowledging that WSLA modules were effective at integrating concepts across subjects. While a better perspective should emerge over time, there are many advantages in moving towards the integration of basic sciences with WSLA. One is the benefit of providing biomedical education with a holistic approach rather than a fragmented one. A better understanding of foundational courses in a clinically relevant context may improve our students’ performance and employability, since concepts from basic disciplines are essential today for understanding and treatment of illnesses. On the other hand, the frustration caused by a basic sciences cycle that does not match the expectations of first-year students has been described as a risk in burnout [26]. With our WSLA instrument we aim not only to better train them in solving clinical cases but also to minimize the burnout effect during early preclinical years by reinforcing basic science knowledge transferred to a clinical context.

However, there are some limitations we should not ignore. WSLA requires a certain critical level of institutional coordination and commitment. Given that WSLA should merge with other learning activities and requires the involvement of different actors, it is very important to plan lectures, practices and WSLA modules at the beginning of each academic year. We have found evidence that some students and teachers may struggle with the new approach. Reluctant teachers trying out WSLA may not actually adopt the practice-based paradigm but instead may use classical teaching methods during the class. Learners who experience WSLA but do not adopt the paradigm tend to feel that they have gained less knowledge [27]. As in any process of change, all these factors should be considered, but the need for more successful integration in medical education is beyond any doubt.

## Conclusions

The WSLA could be a flexible and scalable instrument for moving towards integrated curricula, and can be successfully adapted to teach basic subjects in preclinical years of health science degrees. WSLA can be used with large groups of students and in a variety of contexts or environments using clinical cases as connecting threads. Further research will help to identify additional improvements and to evaluate the impact of this new instrument across several academic years.

## Abbreviations

ANECA: Agencia Nacional de Evaluación de la Calidad y Acreditación
EHEA: European Higher Education Area
ICT: Information and Communication Technologies
iRAT: Individual Readiness Assurance Test
MIR: Medical Internal Resident
tAPP: Team Application Process
TBL: Team Based Learning
UEM: Universidad Europea de Madrid
WSLA: Work Station Learning Activities
